# Incidence of multiple sclerosis in Northern Lisbon, Portugal: 1998–2007

**DOI:** 10.1186/s12883-014-0249-1

**Published:** 2014-12-21

**Authors:** Joao de Sá, Enrique Alcalde-Cabero, Javier Almazán-Isla, Fernando García-López, Jesús de Pedro-Cuesta

**Affiliations:** Neurology Department, Santa Maria Hospital, Av Prof. Egas Moniz, Lisbon, 1600-001 Portugal; National Centre for Epidemiology, Carlos III Institute of Health, Av Monforte de Lemos, 5, 28029 Madrid, Spain; Consortium for Biomedical Research in Neurodegenerative Diseases (Centro de Investigación Biomédica en Red sobre Enfermedades Neurodegenerativas - CIBERNED), Ministry of Economy and Competitiveness, Madrid, Spain; Department Epidemiología Aplicada – CIBERNED, Centro Nacional de Epidemiología, Pab 12, Instituto Salud Carlos III, Av/ Monforte de Lemos 5, 28029 Madrid, Spain

**Keywords:** Capture-recapture, Epidemiology, Methods, Multiple sclerosis, Public health

## Abstract

**Background:**

There are few, recent, well assessed, multiple sclerosis (MS) incidence surveys on European populations. This study sought to measure MS incidence in a Northern Lisbon population and assess it using capture-recapture methods (CRMs).

**Methods:**

Among the population residing in the Northern Lisbon Health Area, registered MS diagnoses were obtained from general practitioners in three primary-care districts covering a population of 196,300, and a neurology unit at the main referral hospital. Cases with onset during the periods 1978–1997 and 2008–2012 were excluded due to perceived poor access to image-supported neurological diagnosis and administrative changes in patient referral respectively. Age- and sex-specific incidences for the period 1998–2007 were calculated using McDonald diagnostic criteria, and CRMs were used to correct age-specific incidence rates. The corrected figures were also adjusted for age using the European Standard Population as reference.

**Results:**

When applied to 62 MS patients with onset in the period 1998–2007, the rates per 100,000 population were as follows for both sexes: crude, 3.16; age-adjusted, 3.09 (95% CI 2.32 to 3.87); CRM-adjusted, 4.53 (95% CI 3.13 to 5.94); and age- and CRM-adjusted, 4.48 (3.54-5.41). In general, the rates were 3-fold higher among women than among men. Negative source dependency and CRM impact were highest at ages 35–44 years, where a 60% rise led to a peak incidence.

**Conclusions:**

MS incidence in Northern Lisbon, Portugal, is moderately lower than that yielded by surveys on European populations. CRMs, which in this instance suggest undercounts, are a potentially useful tool for case-finding assessment but their application may introduce bias.

## Background

The incidence of multiple sclerosis (MS) in Portuguese populations is not known. Prior reports on the occurrence of MS in these populations have been limited to prevalence studies [1]. A recent review of MS incidence surveys conducted in the European Economic Area in the period 1985–2009 revealed that, after 1985, MS incidence ranged from just over 1 to almost 7 per 100,000 population, was higher in females, tripled with latitude, and doubled with midpoint year of study period [2]. Some observations [3,4] suggest that MS incidence in southern Europe, i.e., in Catania 2000–2004 and San Marino 1990–2005, may have reached or even exceeded the magnitude seen for northern European populations. Changes in diagnostic criteria –which are broader now than in the past–, the growing role of neurologists in the management of the disease, the use of more specific laboratory and imaging techniques, and the application of new therapeutic tools, unknown until only just a few years ago, may have all contributed to a keener awareness of the disease among health-care workers and patients alike.

The capture-recapture method (CRM) is a classic procedure intended to estimate prevalence or incidence, by adjusting for the extent of incomplete ascertainment where cases are collected from different sources [5,6]. The results of applying CRMs to MS prevalence have been reported by seven studies, in every case based on crude prevalence counts [7-13], and show that increases in prevalence estimates vis-à-vis uncorrected figures are high, rising to 38% in a recent Spanish study [12]. Although capture-recapture methods have hardly been applied to MS incidence, two studies [13,14] have shown CRMs effects to be stronger, leading to 47.3% and 80% increments in crude incidences respectively.

Hook and Regal have stressed the importance of validating CRMs using "real" data instead of simulated information [15], and recently Jones et al. reported problems in applying CRM to surveys where there had been underlying referral of patients between sources [16]. We explored the feasibility of applying CRM, using age-specific incident MS samples and selecting methodological options deemed to be appropriate for the disease where populations differ in age-structure [17,18]. In sum, CRMs are increasingly used in MS surveys and their impact on measurements is high. However, MS researchers might not have been fully aware of major CRM flaws. Accordingly, this study sought to assess MS incidence in a Portuguese population and apply CRMs to the incidence sample.

## Methods

### Study populations and medical services

**Figure 1:**
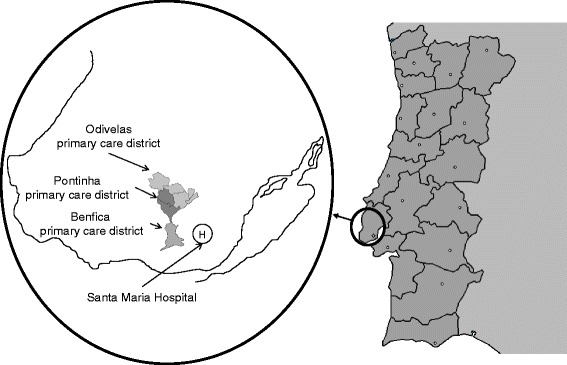
**Geographical location of study populations within the Northern Lisbon area.**

We studied MS incidence during a specific period among the resident catchment population of three, geographically adjacent, primary-care districts in Lisbon's Northern Health Area, i.e., Benfica, Pontinha and Odivelas, which account for an overall, socially mixed population of 196,300, made up of a working-class stratum in Pontinha and middle- and upper-class strata in part of Odivelas and Benfica. These subpopulations are traditionally served by the Santa Maria Hospital (SMH), which also provides neurological care to a population of more than double the size. The geographical situation of the catchment areas of the above three primary-care districts in the early years of this century and the location of the SMH are depicted in Figure 1. In terms of quality, this population's access to neurology specialists matches that described for other urban Portuguese populations.

Neurological care in Portugal, with one neurologist per approximately 30,000 inhabitants, is provided by the country's public National Health Service (NHS), special social health insurance schemes for certain professions (health subsystems), and voluntary private health insurance (see de Sá et al. [18] for details). In Portugal, primary care is provided at NHS primary-care centres (PCCs), with each centre generally serving the catchment population residing in the geographical area in which it is located. As the cost of MS treatment is wholly subsidised by the NHS, MS patients are required to be registered at the health centres in their respective residential areas. Insofar as the study populations were concerned, Lisbon's SMH, where this study's principal researcher (JS) practices, has been the main referral centre for their designated regional health area for decades.

The ongoing economic crisis, which has been in evidence since the mid-2000s, has had an impact on the Portuguese health care administration, potentially modifying referral to neurologists based at public hospitals, MS diagnosis, and the availability of data for applying McDonald diagnostic criteria, which encompass cranial magnetic resonance imaging (MRI). Firstly, the delay in performing a cranial MRI at the SMH, which was approximately one year in the mid-2000s, has inevitably increased since 2008 as a result of general restrictions on performing MRI studies at public health facilities and this, in turn, has resulted in an important delay in MS diagnosis for patients with recent onset. Secondly, in April 2007 a large private hospital was opened in the Northern Lisbon geographical area. It was created to serve patients with private insurance and private health systems. Thirdly, general practitioner (GP) units were also targeted for change: Family Health Units (*Unidades de Saúde Familiares*) were private administrative units created towards the end of 2008 and co-existed alongside the traditional primary care units in the field, such as those in Odivelas, Benfica and Pontinha. This implicated a redistribution of local GPs and, though it did not modify the study population, communication between the publicly-run hospital units, such as the SMH, and these new units may have differed from that which had existed with the traditional PCCs. Fourthly, in 2011 a mixed public/private unit staffed by seven neurologists was set up in Loures, and has since become the reference unit for patients from the Odivelas and Pontinha PCCs. Traditionally, national regulations in Portugal confer a limited capacity on neurologists and other clinicians to provide immunomodulatory treatments to MS patients, in cases where MS patients are not referred to a publicly-run hospital.

### Diagnostic criteria, case-finding and outcome of epidemiological classification

The project was approved by the SMH Research Ethics Committee in 2007. All persons gave their informed consent to JS prior to their inclusion in the incidence study.

#### Diagnostic criteria

Only patients ever fulfilling McDonald 2001 MS diagnostic criteria [19] were included in the case series resulting from case-finding and are referred to as "MS patients". Patients suspected of suffering from MS but still undergoing clinical evaluation or having symptoms difficult to interpret, were not included in the database. Patients fulfilling McDonald criteria for clinically isolated syndrome at their most recent neurological visit were excluded.

#### Case finding

A case search was independently conducted at two sources, namely, PCCs and the SMH.

#### Primary-care-based case search

In 2009, all PCCs in Lisbon's Northern Health Area were invited to participate in this survey. After the directors of the Benfica, Pontinha and Odivelas PCCs presented the project to their staff, all three volunteered to participate. During several meetings with a neurologist field researcher at the three PCCs, GPs received instructions and criteria for identifying patients suffering from MS, i.e., patients were required to present with diagnosis of MS established by a department of neurology or a local neurologist. The GPs agreed to participate and complete a spreadsheet with patient data. At each centre, the Director appointed a local researcher to implement data-collection. All the GPs −57 in Odivelas, 13 in Pontinha and 36 in Benfica- collaborated. Field data-collection at the three centres was undertaken in the period September 2009 to December 2010 and updated in December 2012 to cover cases with potential diagnostic delay. When GP data were furnished to the research team for the purpose of applying diagnostic criteria, JS contacted patients' neurologists who were active at the facilities where the MS diagnoses known to the GPs had been made: these comprised private neurologists reporting to the three PCCs (ten cases) and neurologists serving at publicly-run neurological departments at Lisbon hospitals (i.e., the Capuchos and Egas Moniz Hospitals, eight and five patients respectively). After examining 25 case records, JS excluded five patients for different reasons: three for not fulfilling diagnostic criteria due to dissemination-in-time requirements; and two others for having conditions other than MS (one with MRI lesions not typical for MS, and the other with isolated myelitis with normal cranial MRI).

#### Search based at the Santa Maria Hospital

During the study period, the database of patients diagnosed with MS at the SMH unit directed by JS was explored in order to identify residents in the above three primary-care districts. In addition, a few patients with MS but not shown on the GP lists were identified by JS after a request for information had been sent to neurologists in private practice. Patients were labelled as being known or unknown to the GPs, firstly by reason of the fact that their names were absent from the respective GP's list, and secondly on confirmation at the above-mentioned meetings of the fact that there was no record of MS diagnosis in the patient's clinical history at the PCC. JS examined the neurology records of patients attending the SMH and applied diagnostic criteria at the end of 2010 and again at the end of 2012.

### Choice of incidence study period and ascertainment of residence at clinical onset

**Figure 2:**
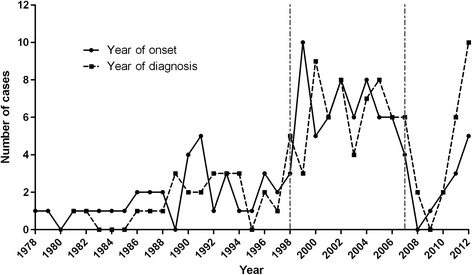
**Outcome of case-finding by year of onset and diagnosis.** Annual number of multiple sclerosis onsets or multiple sclerosis diagnoses for 106 cases fulfilling diagnostic criteria among the pooled population from 1978 to 2012. The study period, 1998–2007, is marked between vertical dash-dotted lines.

**Figure 3:**
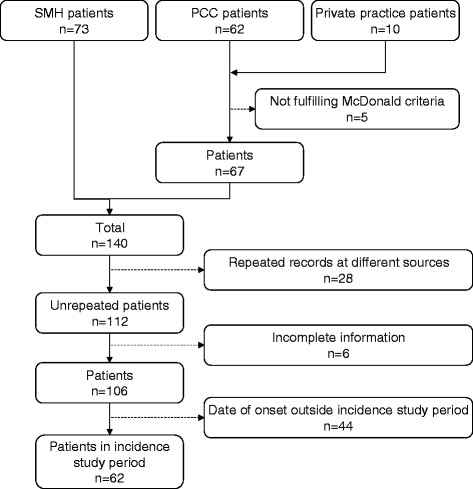
**Study participant flow chart.**

Once the PCC and SMH case-finding had been deemed to be complete, the diagnostic criteria applied and the patients' residence initially identified, the annual number of MS onsets was then tabulated for 112 MS patients and plotted for 106 with known year of and age at clinical onset (see Table 1 and Figure 2). Completion of case-finding by contact with clinicians or managers at the above private facilities which had come into existence since 2008 was considered to be unfeasible. A thorough discussion of the case-finding outcome determined the selection of the survey incidence period as the ten-year interval from 1998 to 2007. The reason was that potential patients with onsets in this period were regarded as having the highest probability of undergoing an MRI study (which became standard practice from 1998 onwards, coinciding with the opening of a specific MS unit at the HSM) and being identified by the case-finding strategy. All MS patients with clinical onset across the period 1998–2007 and listed in Table 1 presented with their first MS symptoms while residing in the catchment area of one of the three PCC districts, as verified from GPs' administrative documents, SMH records or both. An outline of the participant flow from case-finding to becoming MS incidence patients included in the study is shown in Figure 3.

**Table 1:** **Case-finding and distribution of cases fulfilling McDonald diagnostic criteria by age group, sex and year of clinical onset**

**Calendar year**	**Age groups**																	
	**0-14**		**15-24**		**25-34**		**35-44**		**45-54**		**55-64**		**65+**		**Unknown**		**Total**	
	**M**	**W**	**M**	**W**	**M**	**W**	**M**	**W**	**M**	**W**	**M**	**W**	**M**	**W**	**M**	**W**	**M**	**W**
1978								1										1
1979			1														1	
1980																		
1981						1												1
1982						1												1
1983								1										1
1984					1												1	
1985								1										1
1986					1	1											1	1
1987							1	1									1	1
1988			1			1											1	1
1989																		
1990		1				2		1										4
1991			2			2				1							2	3
1992						1												1
1993			1		2												3	
1994										1								1
1995								1										1
1996							2	1									2	1
1997							1	1									1	1
1998						1	1	1									1	2
1999			2		1			5		2							3	7
2000			1	1				2						1			1	4
2001				3			1	2									1	5
2002					1	2	1	3				1					2	6
2003			1	1		2	1			1							2	4
2004			1	1	1	1		1		3							2	6
2005				1	1			3				1					1	5
2006				1		2		3										6
2007						3		1										4
2008																		
2009						1												1
2010				1		1												2
2011				1				2										3
2012						2		3										5
Unknown															2	4	2	4
Total		1	10	10	8	24	8	34		8		2		1	2	4	28	84

M indicates men; W indicates women.

### Denominators of incidence counts

For the purpose of generating age-specific populations close to the mid-point of the incidence period, we obtained age-specific 2001 census populations for administrative units that overlapped the residential areas of primary-care users (i.e., those of the Benfica and Pontinha parishes and the Odivelas municipal area) and yielded a reasonable fit with primary-care districts of the same name (here denoted as the "Northern Lisbon Districts population"). Male and female catchment populations for each of the above-mentioned PCCs in the mid-2000s were obtained from the health authorities, and estimates of age and sex distributions were drawn up using the above-mentioned 2001 census data. The distributions of each of the three health-district populations by age and sex are shown in Table 2.

**Table 2:** **Average population and time at risk during incidence period (1998–2007), as well as age-specific and overall crude and adjusted incidence rates**

	**Person-years**			**Cases**			**Incidence rates**		
**Age group**	**Men**	**Women**	**Total**	**Men**	**Women**	**Total**	**Men**	**Women**	**Total**
0-14	134,440	128,500	262,940	0	0	0	0.00	0.00	0.00
15-24	143,300	138,040	281,340	5	8	13	3.49	5.80	4.62
25-34	154,460	149,600	304,060	4	11	15	2.59	7.35	4.93
35-44	124,980	133,820	258,800	4	21	25	3.20	15.69	9.66
45-54	136,010	158,730	294,740	0	6	6	0.00	3.78	2.04
55-64	127,850	140,930	268,780	0	2	2	0.00	1.42	0.74
65+	119,570	172,890	292,460	0	1	1	0.00	0.58	0.34
All ages	940,610	1,022,510	1,963,120	13	49	62	1.38	4.79	3.16
Adjusted rates							1.30 (0.59-2.01)^a^	4.79 (3.44-6.13)^a^	3.09 (2.32-3.87)^b^

^a^Age-adjusted and ^b^age-and sex-adjusted (European population) with their corresponding 95% confidence intervals.

### Capture-recapture methodology

#### Data-source design

**Figure 4:**
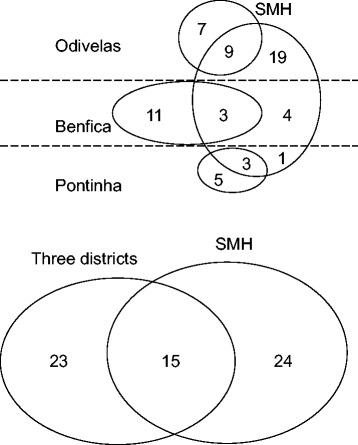
**Distribution of multiple sclerosis patients with onset in the period 1998–2007, by main source, primary-care district (Odivelas, Benfica and Pontinha) and SMH, Santa Maria Hospital.**

In accordance with early procedural suggestions [6,20], patient identification was followed by distinguishing three "original" sources, namely, the SMH, three GP lists at PCCs, and some private practitioners; and two "analytical" sources, the SMH and PCCs, used for CRM calculations, after private practitioners and GPs at PCCs had been pooled into one source. Independence of data sources was verified by testing the statistical significance of deviations between the expected and observed distribution of cases at the SMH and PCC intersect on a 2×2 table, obtained from product probabilities of the main analytical subsets and shown in Figure 4.

#### Calculations for CRM

**Figure 5:**
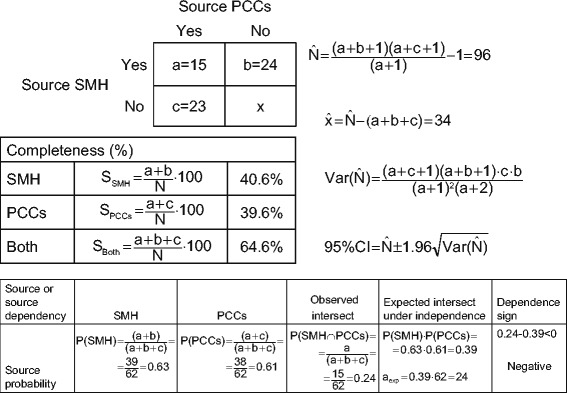
**Calculation of correction by recapture, completeness and source dependency on multiple sclerosis incident patients during the period 1998–2007.** PCC indicates primary-care centre; SMH, Santa Maria Hospital; and S, sensitivity (or completeness).

We proceeded as summarised in Figure 5, assuming source independence on the basis of reported methods [6,21-24]. CRM-corrected incidence estimates and their 95% confidence intervals (CIs) were obtained for pooled populations at all ages and by age-specific strata. To recapitulate, the CRM-corrected incidence numerator was generated with the Sekar and Deming method [21], as modified by Chapman [22] and Seber [23], using the procedure described in Hook and Regal [15] and termed the Chapman nearly unbiased estimator (NUE), which is shown in Figure 5 with an example for all districts across all ages. The estimated value for the unobserved cell was obtained, as indicated in Figure 5, from the rounded Chapman NUE figure minus the three observed cell numbers. The 95% confidence interval (CI) of CRM-corrected incidence was obtained from 95% limits for the CRM-corrected value, by using the procedure for the Chapman NUE variance calculation [22,23] as suggested by Ferrer Evangelista et al. and Gallay et al. [25,26] (Figure 5). Finally, CRM-corrected and -uncorrected incidence figures and 95% CIs were age-adjusted by the direct method, in order to compare them to virtual rates from a standard population. We used European Standard Population weights provided by Zivadinov *et al.* [17] for MS incidence strata [17], and the Epidat 3.1 software procedure (Galician Regional Authority, Santiago de Compostela, Spain/Pan American Health Organisation, Washington, D.C, 2005) for direct adjustment.

#### Completeness

Heterogeneity of completeness (captured proportion of estimated cases, i.e., ratio of cases detected by a given source to total estimated cases) was tested using the *χ*^2^ test for 2×2 or 2×n contingency tables.

### MS incidence comparisons with other surveys

For the purposes of interpreting results, the age-specific MS incidence in Northern Lisbon, both CRM-corrected and -uncorrected, was compared to age-specific MS incidence as reported by three, recent, selected surveys that were undertaken in Catania (Italy) [4], Lorraine (France) [27] and Västerbotten (Sweden) [28] using the less sensitive Poser clinical or clinical and laboratory supported criteria for probable MS [2].

## Results

As briefly mentioned under Methods, after detailed examination of neurology records kept by the SMH and a few specialists (mostly neurologists) contacted by GPs and JS, 62 patients were found to fulfil McDonald 2001 criteria for MS, with clinical onset during the period 1998–2007 and residence in Northern Lisbon's three PCC districts.

The 62 MS patients classified by source, original or analytical, are depicted in Figure 4. Balance by origin was in evidence, with 24 cases seen exclusively at the SMH, 23 seen exclusively at PCCs and 15 seen at both sources. During the period 1998–2007, overall crude incidence (Table 3) was 3.16 and age-and sex-adjusted incidence was 3.09 per 100,000 person-years (95% CI 2.32 to 3.87). Crude and age-adjusted MS incidence per 100,000 person-years was 1.38 and 1.30 (95% CI 0.59-2.01) among men, and 4.79 and 4.79 (95% CI 3.44 to 6.13) among women respectively. When calculated for 38 PCC patients, crude MS incidence per 100,000 person-years was 1.94, and when calculated for 39 SMH patients it was similar, namely, 1.99 (data not shown in table), and considerably lower than two-source incidence. Peak values for age-specific figures were seen at ages 35–44 years, with the figure for both sexes being 9.66 per 100,000 person years and that for women being 15.69, based on 21 cases; the figures for men were unstable, though no cases started after the age of 44 years. Based on the figures shown in Figure 5 and Table 3, in which the data are broken down by age and source, the CRM-corrected number of cases (N) was 96. Completeness (Figure 5) for SMH and PCCs across all ages was 40.6% and 39.6% respectively, rising to 64.6% for both sources combined. Source probability for SMH and PCCs across all ages was 0.63 and 0.61 respectively. The expected intersect, 0.63 × 0.61 = 0.39, was higher than the observed intersect 15/62 = 0.24, with the dependency sign (0.24-0.39 < 0) thus being negative. Observed vs. expected numbers at the intersect, 15 vs. 24, and outside the intersect, 47 vs. 38, yielded an odds ratio = 0.50 (95% CI 0.21 to 1.17).

**Table 3:** **Age distribution of incident MS patients for 1998–2007: observed cases (OC) and estimated cases (EC) as well as percentage of completeness**

		**No. of patients**									**Incidence rates per 100,000**		
		**OC**					**Completeness%**				**Crude**	**Ascertainment corrected**	
		**SMH**	**GP**	**Both**	**EC**	**Total**	**SMH**	**GP**	**Both**	**Chapman correction**	**Crude**	**Ascertainment corrected**	**Age-adjusted point**
											**Point**	**with 95% CI**	**with 95% CI**
**Age group**	**Person-years**												
0-14	262,940	0	0	0	-	-	-	-	-	0.000	0.00	-	-
15-24	281,340	6	3	4	3	16	62.5	43.8	81.3	16.600	4.62	5.69 (3.42-7.95)	-
25-34	304,060	4	6	5	4	19	47.4	57.9	78.9	19.000	4.93	6.25 (4.07-8.43)	-
35-44	258,800	13	7	5	15	40	45.0	30.0	62.5	40.167	9.66	15.46 (8.30-22.61)	-
45-54	294,740	1	5	0	5	11	9.1	45.5	54.5	11.000	2.04	3.73 (0.09-7.37)	-
55-64	268,780	0	1	1	0	2	50.0	100.0	100.0	2.000	0.74	0.74 (0.74-0.74)	-
65+	292,460	0	1	0	0	1	0.0	100.0	100.0	1.000	0.34	0.34 (0.34-0.34)	-
All ages	1,963,120	24	23	15	27	89	43.8	42.7	69.7	89.767	3.16^a^	4.53 (3.13-5.94)^b^	4.48 (3.54-5.41)^d^
All districts	1,963,120	24	23	15	34	96	40.6	39.6	64.6	96.500	3.16^a^	4.89 (3.49-6.29)^c^	-

MS: multiple sclerosis; OC: observed cases; EC: estimated cases; SMH: Santa Maria Hospital; GP: general practitioners; CI: confidence interval.

^a^Crude estimates; ^b^ascertainment-corrected incidence obtained from added age-specific N = 89 estimates; ^c^ascertainment-corrected incidence obtained from crude N = 96 estimates; ^d^population age-adjusted and capture-recapture corrected rates.

Shown in two sections, upper and lower, in Table 3 is the 2×2 breakdown of the two-source model when applied to nine observations, seven of which correspond to horizontally aligned age-specific strata for pooled districts (upper), and two of which correspond to the pooled districts across all ages (bottom). The second last row contains totals for all ages from vertically aligned age-strata. The body of the table is divided into a left block with cases and completeness results, and a right block with incidence figures, showing crude, age-specific, uncorrected or CRM-corrected, and age- and CRM-adjusted estimates.

The left block of Table 3 shows both the observed data and the rounded Chapman NUE numbers, which was 89.8 for all districts at all ages, and ranged from one for the 65-and-over to 40 for the 35–44 group on an age-specific basis. When NUE estimates for age groups were added, the rounded number, namely, 90, was 7% lower than the figure of 96 obtained when the method was applied to crude pooled populations. While the CRM-estimated number of patients at ages ≥65 years was low, i.e., one, it exceeded 40 in the 35–44 age group. Completeness for two sources and all ages was 64.6%. Two-source completeness for age-specific groups was >78%, except for the 35–44 and 45–54 age groups, but did not prove significantly heterogeneous, with Fisher's exact test *p* = 0.441, Pearson ${\chi}_5^2 = 5.258$.

Incidence per 100,000 person-years in age-groups shown in Table 3 (bottom right) displayed a considerably wide variation, ranging, after correction for ascertainment, from 0.34 at age 65 years and over to 15.46 at ages 35–44 years. CRM impact was highest for the 35–44 age group, increasing from an observed 9.66 per 100,000 person-years to a CRM-adjusted 15.46 per 100,000 person-years, i.e., by 60%. Incidence at ages 45–54 years and over compared to that at ages 30–44 years was low, but the CRM impact among the 45–54 age group was non-negligible, i.e., 83%, based on small numbers, and nil at ages 55 years and over, with a limited effect on overall CRM impact in the age-adjusted measurements. Finally, a complete overview of MS incidence is shown in Table 3, for which crude observed incidence per 100,000 person-years was 3.16, increasing after CRM correction to 4.53 per 100,000 person-years, i.e., by 43%, and after additional age-adjustment to 4.48 per 100,000 person-years, i.e., by 42%.

**Figure 6:**
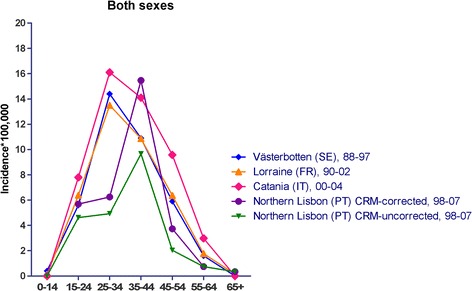
**Age-specific multiple sclerosis incidences reported for selected surveys, including those seen in Northern Lisbon before and after capture-recapture correction.** CRM indicates capture-recapture method.

Figure 6 depicts age-specific MS incidence in Northern Lisbon, both CRM-corrected and -uncorrected, as well as the same reported rates in Catania [4], Lorraine [27] and Västerbotten [28]. With regard to the uncorrected figures, it would appear that considerably lower rates were seen for the 25–34 year age-group, with those for the other age groups being moderately lower. CRM-corrected incidence at ages 35–44 years exceeded that reported by other surveys.

## Discussion

In addition to providing detailed figures of MS incidence in a Northern Lisbon population, which is similar to or moderately lower than that in other European populations, this study shows that age- and CRM-adjustment considerably modify crude figures, that the impact of CRM adjustment can be age-specific, and that adjustment for age has a small impact, given the coincidence of the age structure of the European Standard and Portuguese study populations.

A key question to be answered is the validity of our CRM-corrected measurements and the significance of CRM. Capture-recapture methods have been widely discussed and some authors have suggested a need for caution when using them in epidemiology [16,29-32]. In contrast to expected CRM results, namely, that most data sets used by epidemiologists tend to have a net positive dependence [15], our results suggest that the source dependency present in our study was negative, and highest for the 35–44 age-group, something that would tend to produce CRM-corrected incidence overestimates and thereby reduce the validity of the application [6]. Negative dependency, namely, a situation in which the proportion of cases known both to the SMH and PCCs is too low, applies here because the number of cases, 15, captured by the SMH and PCC intersect is too low compared to the expected proportion of 39%, i.e., 24 cases. According to our expectations, and contrary to what was anticipated by Jones et al. [16] in the presence of referrals between sources, the positive dependency due to the fact that patients with MS attending PCCs have historically tended to be diagnosed at the SMH as a result of traditional referral policies and geographical proximity, was neutralised or even reversed. We believe that negative dependency in our series might point to unnoticed losses in case-finding of MS patients who, rather than being sourced at the SMH as expected, were instead missing. Negative dependence was highest in the 35–44 age group, which is difficult to explain. All things considered, CRM-corrected results would seem to be overestimated, while uncorrected results, in contrast, might constitute underestimates because of limited case finding. We believe that the most accurate and valid measurement of MS incidence in the Northern Lisbon Districts population is closer to the CRM-uncorrected age-specific measurements, thus reinforcing the notion of similar or moderately lower incidences.

For comparisons between surveys, our uncorrected measurements point to incidences at ages <24 and >35 years which are almost similar to those of the Catania, Lorraine and Västerbotten studies, whereas incidences at 25–34 years are atypically lower than expected. Assuming that MS incidence in Portugal behaves as in other northern or southern European populations, among whom incidence peaks in the 25–34 age-group [2], the notions of a switch from age-at-onset towards age-at-diagnosis in our survey, possibly attributable to a behavioural pattern in seeking care, would also have to be considered. The Catania, Lorraine and Västerbotten studies used Poser diagnostic criteria, which included laboratory supported criteria (perhaps 20% more sensitive) in the case of Catania and Västerbotten. McDonald criteria established MRI as the standard for capturing disease dissemination [33], enabling a definite MS diagnosis to be established 12 months earlier where a cranial MRI disclosed a new T2 lesion, as shown by Tintoré et al. [34]. It would thus appear that part of the difference between selected surveys could be attributed to diagnostic criteria, and that the difference in incidences with respect to the Lisbon survey was masked by use of more sensitive diagnostic criteria. The expected effect of the midpoint of the incidence period on differences is difficult to assess. When using a model fitted in a prior study [2] with adjustment for age and sex, our CRM uncorrected incidence shows a lower rate ratio (0.55 with a 95% CI 0.40-0.74) than that in Lorraine, together with an absence of significant differences when compared to those in Västerbotten, Catania and our CRM-corrected incidences, yielding a rate ratio of 0.77 (and a 95% CI 0.59-1.01) for the latter. These figures may support a dominant negative bias effect of limited case finding which would prevail over the positive effect of diagnostic criteria. The long duration of the period 2008–2012 affords protection against the effect of diagnostic delay [35], with our overall incidence thus being only moderately lower than in other French, Italian or Nordic populations.

New health policies introduced in Portugal as from 2008 led to changes in referral from the study areas to publicly-run hospitals. This prevented accurate ascertainment of any incident cases occurring after that date, and so our study had to be limited to the period from 1998 to 2007. Potential incomplete ascertainment of incident cases during the period 1998–2007 might thus have occurred. One cannot rule out the possibility that a few patients might have moved away from the designated study districts after MS onset and, as a result, would not appear on the collaborating GPs' lists. Similarly, patients with mild MS symptoms may not have sought after-care or been referred to neurologists. Furthermore, some patients residing in the study region could have been attended by doctors from other regions, thereby leading to an underestimated incidence rate. However, as the cost of MS treatment is wholly subsidised by the country's National Health Service, patients are required to be registered at the health centres in their respective residential areas. This raises the possibility that untreated new MS patients might not be covered by the study. All the above-mentioned factors point to an underestimation of the incidence rate. On the other hand, a few incident cases recorded in the 1998–2007 period might have actually experienced disease onset beforehand, if vague or poorly-defined first MS symptoms had been overlooked at a time when access to MRI was limited. The strengths of our study reside in its use of the more sensitive McDonald MS diagnostic criteria validated by a neurologist specialised in MS, the use of CRC methods to assess the meaning and significance of incidence rates calculated by standard methods, and the priority given to avoiding the effects of changing health care policies by limiting the study period to 1998–2007.

## Conclusions

MS incidence in the Northern Lisbon population is similar to or moderately lower than that in other European populations. CRM, subject to assessment of data-source dependency and applied to patient or population samples, may constitute a potentially useful tool for assessment of the accuracy of case-finding.

## Abbreviations

CI: Confidence interval
CRM: Capture-recapture method
GP: General practitioner
MRI: Magnetic resonance imaging
MS: Multiple sclerosis
NHS: National Health Service
NUE: Nearly unbiased estimator
OC: Observed cases
PCC: Primary-care centre
SMH: Santa Maria Hospital
